# Supplementary material for: The Anti-Tumor Effect of Lactococcus lactis Bacteria-Secreting Human Soluble TRAIL Can Be Enhanced by Metformin Both In Vitro and In Vivo in a Mouse Model of Human Colorectal Cancer
Source: Cancers (Basel). 2021 Jun 15;13(12):3004. doi: 10.3390/cancers13123004 (PMC8232584; doi:10.3390/cancers13123004)
Supplement: Supplementary file 1 [file cancers-13-03004-s001.zip › cancers-1208104-supplementary.pdf]

Supplementary Materials

The Anti-Tumor Effect of *Lactococcus lactis* Bacteria-Secreting Human Soluble TRAIL Can Be Enhanced by Metformin Both In Vitro and In Vivo in a Mouse Model of Human Colorectal Cancer

Katarzyna Kaczmarek, Jerzy Więckiewicz, Kazimierz Węglarczyk, Maciej Siedlar and Jarek Baran

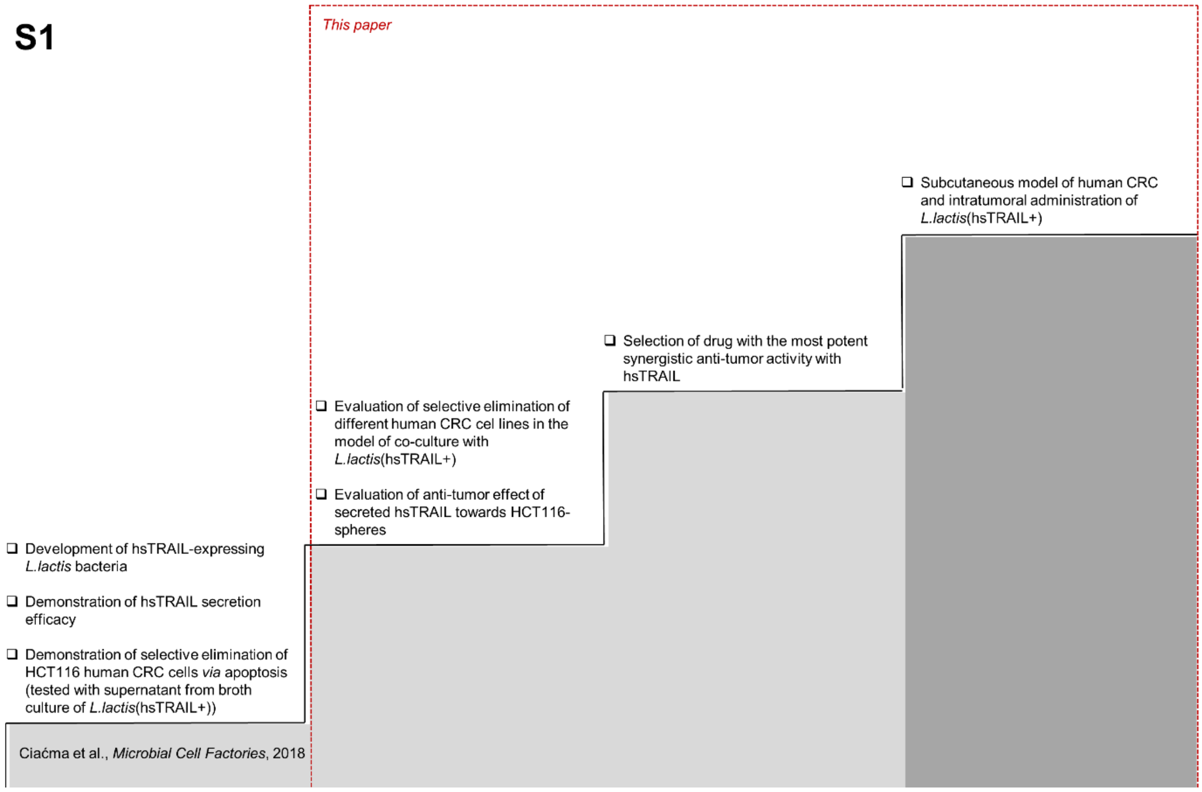

Figure S1. Flow-chart indicating particular stages of the study, including most relevant tasks.

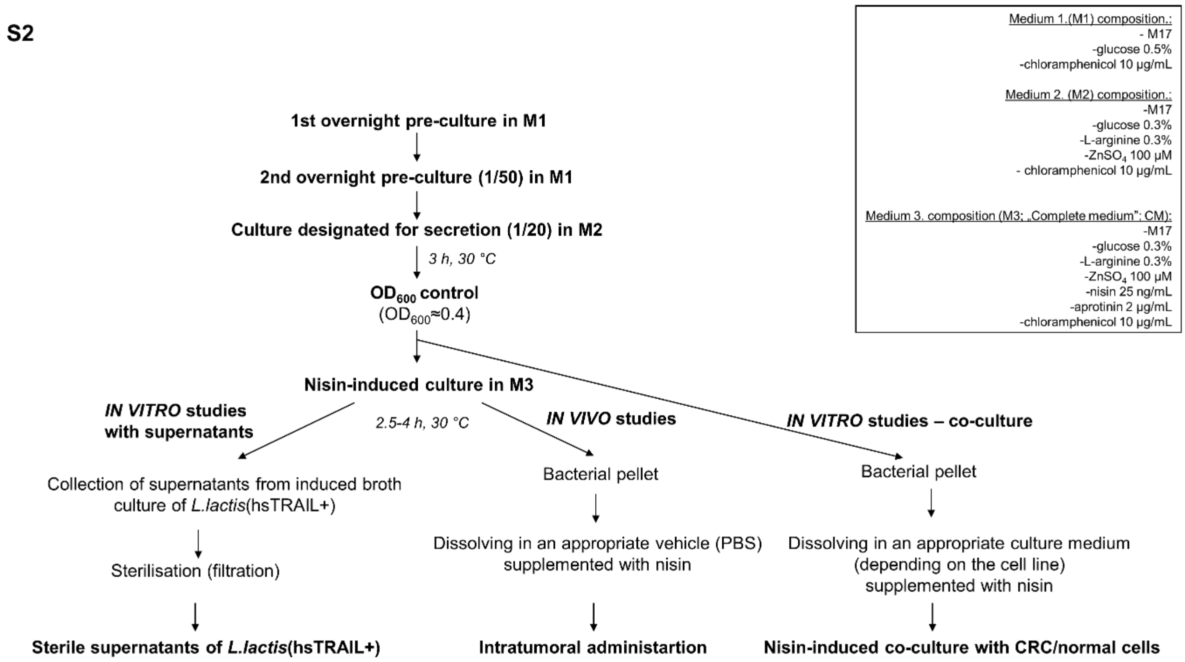

Figure S2. Optimized growth conditions for the developed *L.lactis* (hsTRAIL+) bacteria, based on the nisin-controlled gene expression system (NICE®).

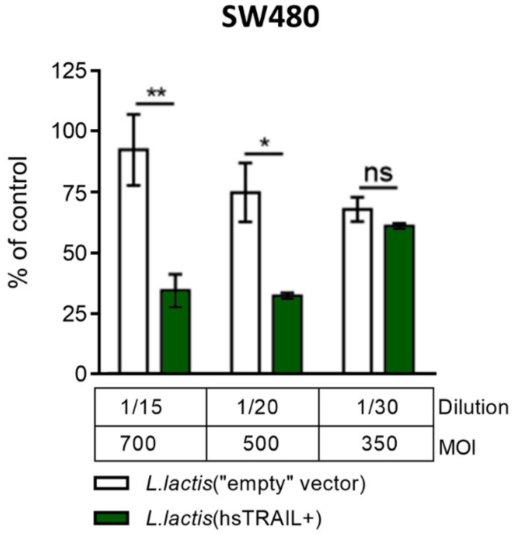

Figure S3. Cytotoxicity of *L.lactis*(hsTRAIL+)-derived hsTRAIL against SW480 cells in a direct co-culture model of cancer cells with bacteria. Human SW480 cells were cultured for 48 h in the presence of *L.lactis*(hsTRAIL+) or corresponding control bacteria (*L.lactis*("empty" vector)) with addition of nisin (inducer). Viability of cancer cells was assessed by MTS test. Results are showed as % of viability of cells incubated in a standard culture medium (y-axis) without bacteria. Legend: MOI - Multiplicity of Infection – the number of bacteria per single eukaryotic cell; 1/15 - 1/30 - dilution of *L.lactis*(hsTRAIL+)/*L.lactis* ("empty" vector) bacteria culture. The bars indicate the mean value ± SEM of two independent experiments, each performed in triplicates. Statistical significance was calculated using two-way ANOVA test, with Tukey's multiple comparisons post-hoc test. * $p < 0.05$, ** $p < 0.01$.

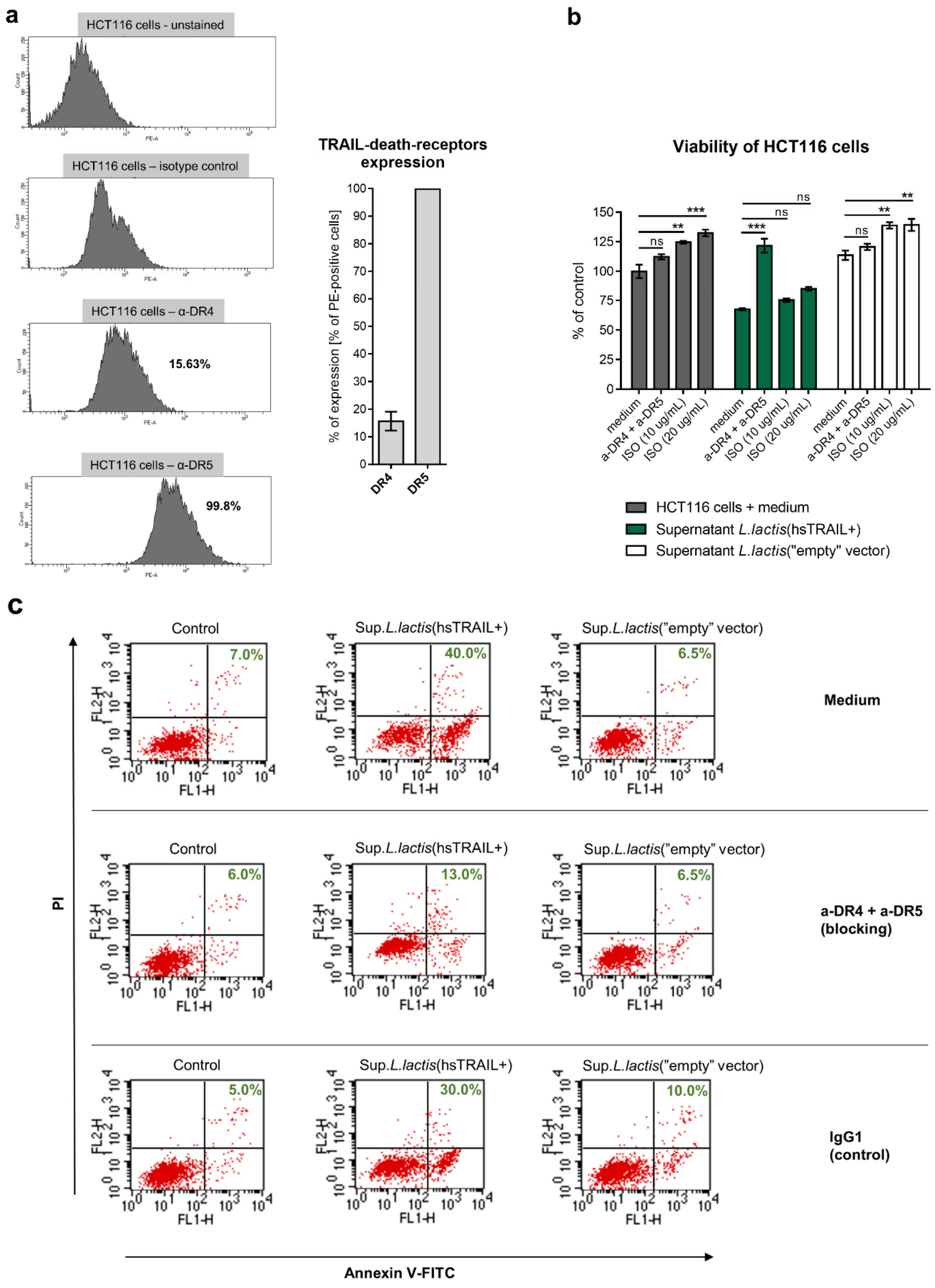

Figure S4. Anti-tumor activity of *L.lactis*(hsTRAIL+)-derived hsTRAIL is mediated by its death receptors: (**a**) Expression of TRAIL-death-receptors DR4 and DR5 on the surface of HCT116 cells evaluated by flow cytometry, using the PE-labelled mouse monoclonal antibodies against human TRAIL receptor DR4 (clone DJR1; eBioscience™, Waltham, MA, USA) or DR5 (clone DJR2-4(7-8); eBioscience™), both at the concentration of 0.25 µg/sample. On the left: representative histograms are shown (*n* = 6); on the right: expression of DR4 and DR5 receptors shown as % of HCT116 PE-positive cells; (**b**) HCT116 cell viability after blocking the TRAIL receptors.

Blocking of DR4 and DR5 significantly reduced the cytotoxic activity of hsTRAIL. HCT116 cells were pre-incubated for 1 h with blocking mouse monoclonal antibodies specific for human DR4 (10 µg/mL, clone HS101; Novus Biologicals, USA) and DR5 (10 µg/mL, clone HS201; Novus Biologicals), before the cells were further treated with concentrated supernatant from *L.lactis*(hstRAIL+) at a dilution corresponding to hsTRAIL concentration of 50 ng/mL; control supernatant (from *L.lactis*("empty" vector) bacteria); medium, for another 48 h. As negative control, an appropriate mouse monoclonal control IgG (clone MG1, Novus Biologicals) was used at the concentration of 10 µg/mL or 20 µg/mL (in relation to blocking of both TRAIL-death-receptors, each at the concentration of 10 µg/mL). Viability of HCT116 cells was assessed by MTS assay. Results are shown as % of viable cells incubated in the standard culture medium or the antibodies used for blocking. The bars indicate the mean value ± SEM of three independent experiments. Statistical significance was calculated using two-way ANOVA test, with Tukey's multiple comparisons post-hoc test. * $p < 0.05$, ** $p < 0.01$, *** $p < 0.001$; (**c**) Apoptosis of HCT116 cells after blocking of TRAIL receptors. HCT116 were pre-incubated with DR4 and DR5-blocking antibodies (each 10 µg/mL), isotype control (20 µg/mL) or medium, as described above. After 48h of treatment with concentrated supernatant from *L.lactis*(hsTRAIL+) bacteria culture at the dilution corresponding to 50 ng/mL of hsTRAIL, or equal volume of concentrated supernatant from the control bacteria, the percentage of apoptotic cells was assessed by staining with Annexin V-FITC and PI (FITC-Annexin V Apoptosis Detection kit, BD Pharmingen, USA), using flow cytometry. The percentage of apoptotic cells (Annexin V+/PI− and Annexin V+/PI+) is shown in the upper right corner of each dot-plot. (**a**) methodology: HCT116 cells (10^6 cells/sample) were resuspended in PBS supplemented with 1% BSA (Sigma Aldrich) and stained with phycoerythrin (PE) labelled mouse monoclonal antibodies against human TRAIL receptor R1/DR4 (clone DJR1; eBioscience™) or TRAIL-R2/DR5 (clone DJR2-4(7-8); eBioscience™), both at concentration of 0.25 µg/sample. As negative control, the cells stained with mouse isotype control IgG1κ-PE (eBioscience™) were used. The cells were stained for 30 min in the dark at RT, then washed (400 × *g*, 5 min, RT), resuspended in 300 µl PBS with 1% BSA and analyzed in a LSR II flow cytometer (BD Biosciences) using the FlowJo software version 10.5.3 (BD Biosciences, USA). The obtained data were analyzed in relation to isotype control. (**b**) methodology: HCT116 cells were seeded onto flat-bottom 96-well plates (Sarstedt) at a density 10^4 cells/well in appropriate culture media, w/o antibiotics. After 20 h of culture in a 37 °C humified atmosphere with 5% CO_2, the media were replaced with fresh growth medium containing 10 µg/mL of mouse monoclonal TRAIL-R1/DR4 (clone HS101; Novus Biologicals, USA) and mouse monoclonal TRAIL-R2/DR5 (clone HS201; Novus Biologicals). As a negative control, mouse monoclonal IgG1 isotype control was used (10 µg/mL and 20 µg/mL, clone MG1; Novus Biologicals). After 1h of blocking, the concentrated supernatant from *L.lactis* (hsTRAIL+) bacteria at a dilution corresponding to 50 ng/mL of hsTRAIL, or from control bacteria at the equal volume, was added. The incubation was continued for another 48h, followed by analysis of cell viability by MTS.

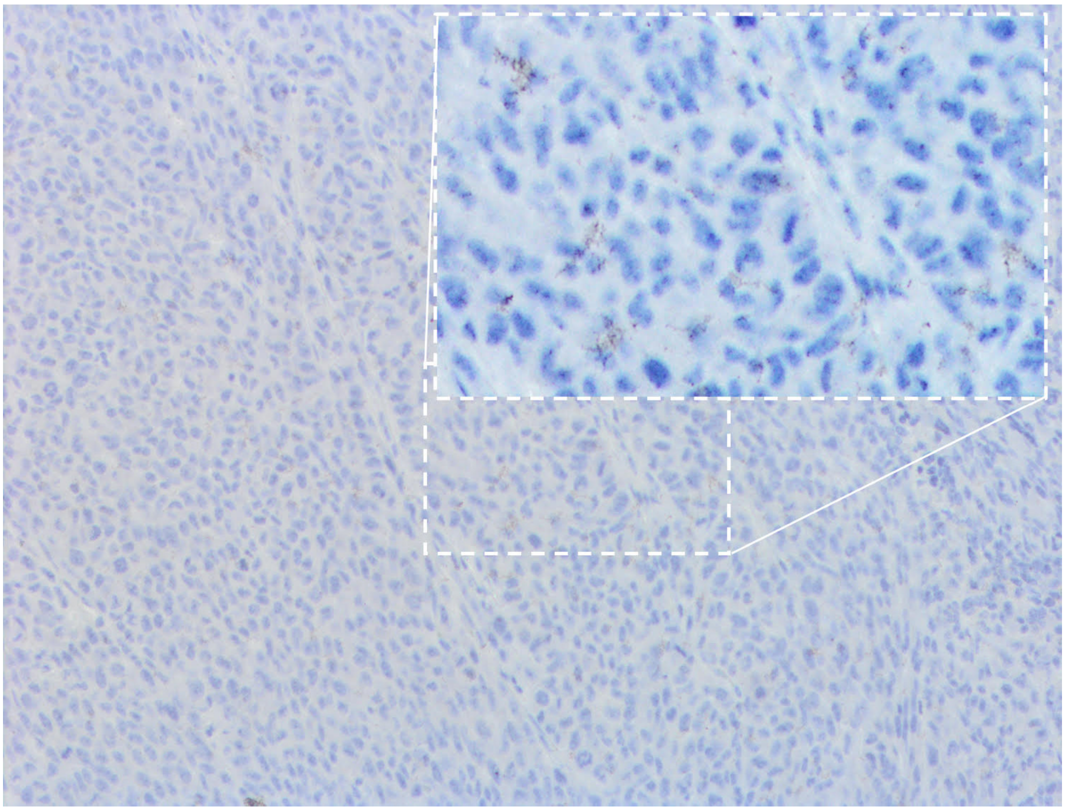

Figure S5. Representative IHC staining of HCT116-tumors, for identification of hsTRAIL secretion after the treatment with *L.lactis*(hsTRAIL+) bacteria (related to Figure 6c in the main text).

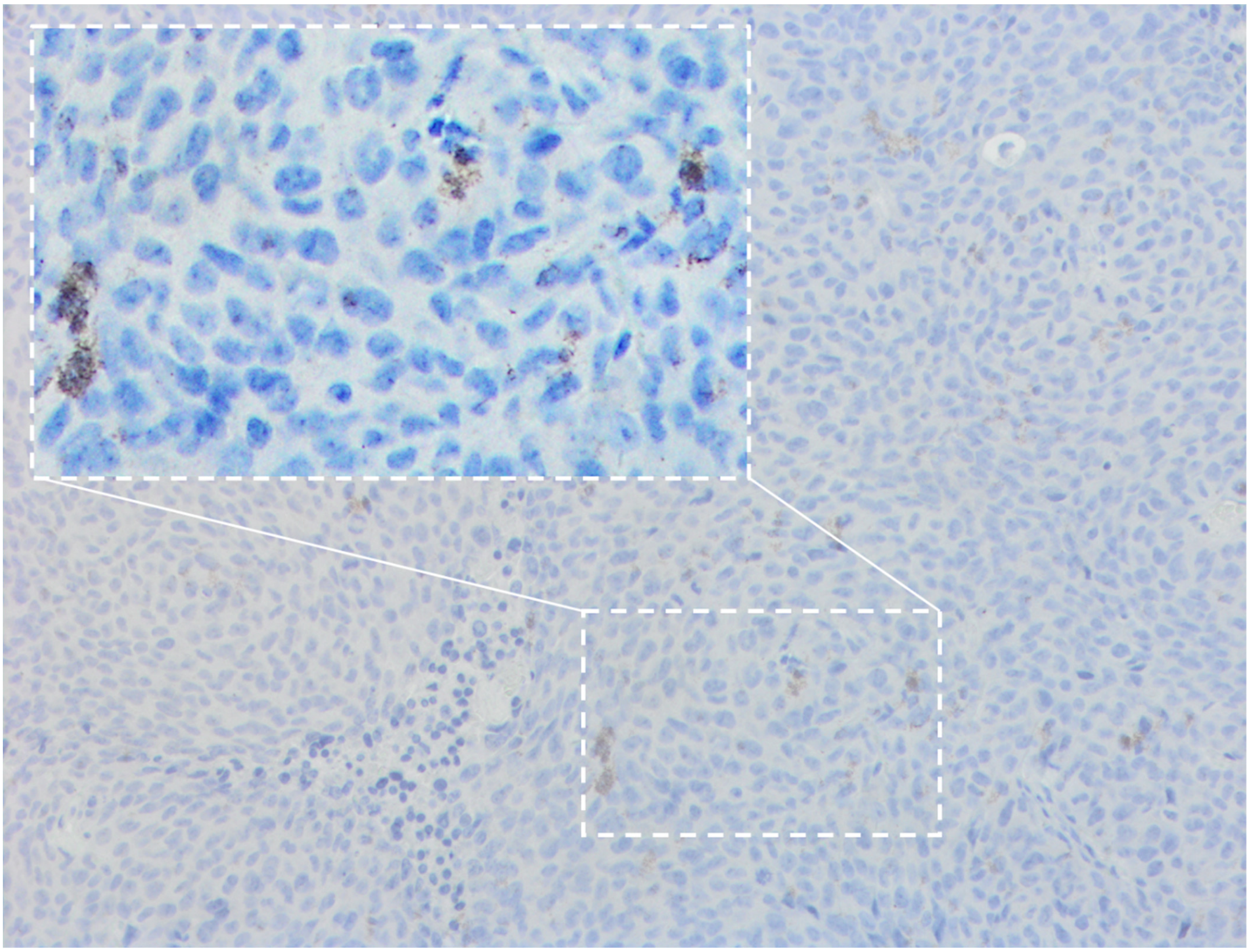

Figure S6. Representative IHC staining of HCT116-tumors, for identification of hsTRAIL secretion after the treatment with MetF + *L.lactis*(hsTRAIL+) bacteria (related to Figure 6c in the main text).

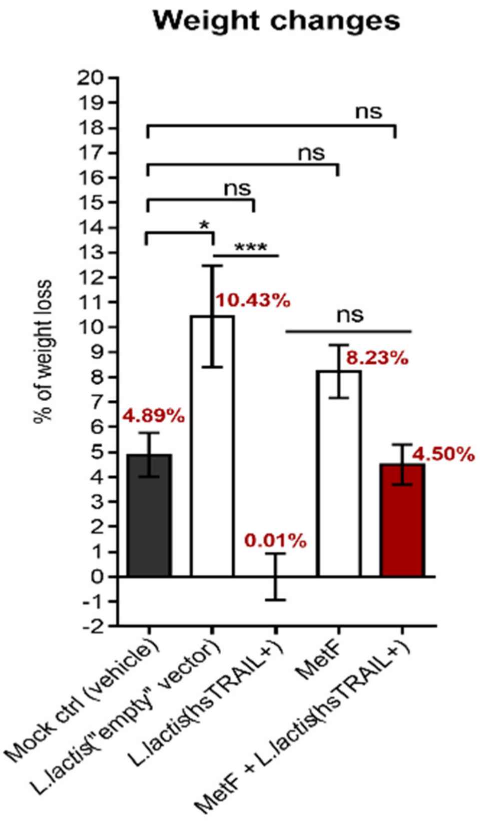

Figure S7. Safety of the intratumor treatment with *L.lactis* bacteria expressing hsTRAIL. Potential toxicity of the specific treatment was assessed by regular measurements of the animal body weight during the study. The figure shows analysis of the loss of animal weight in different treatment groups, performed by comparison of the body weight at the beginning and at the end of the study. The biggest mean weight loss of 10.43% and 8.23% of the initial weight, was observed in the groups of animals receiving intratumor injections of *L.lactis*("empty" vector) and gastric gavages of MetF, respectively. The smallest mean weight loss (0.01%) was observed in the group of mice receiving *L.lactis*(hsTRAIL +), while in the mock control group it was 4.89%. Legend: y axis -% of the weight loss; x axis – experimental group (treatment); red % values - the mean weight loss. The bars indicate the mean value ± SEM. Statistical analysis was performed using the one-way ANOVA test, with post-hoc multiple comparisons using Tukey's method. ** p <0.01.
